# The Moderating Role of Deviant Peer Affiliation in the Relation between Cyber-Victimization, Tobacco and Alcohol Use, and Age Differences

**DOI:** 10.3390/ijerph18168294

**Published:** 2021-08-05

**Authors:** Xiaojun Sun, Liangshuang Yao, Gengfeng Niu, Shanyan Lin

**Affiliations:** 1 Key Laboratory of Adolescent Cyberpsychology and Behavior (CCNU), Ministry of Education, School of Psychology, Central China Normal University, Wuhan 430079, China; sunxiaojun@mail.ccnu.edu.cn (X.S.); ylsccnupsy@126.com (L.Y.); 2 Collaborative Innovation Center of Assessment Toward Basic Education Quality, Central China Normal University Branch, Wuhan 430079, China; 3 Center for Research on Internet Literacy and Behavior, Central China Normal University, Wuhan 430079, China; 4 Department of Psychology, University of Turin, 10124 Torino, Italy; shanyan.lin@unito.it

**Keywords:** cyber-victimization, tobacco and alcohol use, deviant peer affiliation, age difference

## Abstract

Cyber-victimization, tobacco and alcohol use are all prominent public health problems among adolescents throughout the world. Against this background, this study examined the association between cyber-victimization and tobacco and alcohol use, as well as the moderating role of deviant peer affiliation and the potential age differences among elementary, middle, and high school students. A survey conducted among 1488 school students (aged 9–19 years, consisting of 702 elementary school students, 318 middle school students, and 468 high school students) found that cyber-victimization was positively correlated with tobacco and alcohol use among students of all stages. However, the moderating mechanism was different. Among elementary school students, deviant peer affiliation played a positive moderating role. For individuals with high deviant peer affiliation, this association was stronger. Among middle school students, the moderating role of deviant peer affiliation was insignificant. Among high school students, deviant peer affiliation played a negative moderating role; this association was significant for individuals with low deviant peer affiliation. The results of this study clarify the relationship between cyber-victimization and tobacco and alcohol use by examining the moderating role of deviant peer affiliation and age differences, providing intervention guidance for reducing the negative influences of cyber-victimization on children and adolescents with respect their use of tobacco and alcohol.

## 1. Introduction

In our current information society, the internet has become a significant environmental factor influencing the development of children and adolescents. Cyber-victimization refers to the phenomenon whereby an individual is repeatedly bullied by other individuals or groups using electronic communication tools [1]. This is a new but prominent social problem among children and adolescents in recent years. Being cyberbullied may result in many negative outcomes including both internalizing and externalizing problems such as substance use [2,3,4].

Substance use is a prominent public health concern among children and adolescents. In China, it specifically refers to the use of tobacco and alcohol (due to their relatively high prevalence) [5]. Although the sale of cigarettes and alcohol drinks to minors or juveniles is strictly prohibited in China, the phenomenon of smoking and drinking among minors has never disappeared, and recently has even shown a trend of increasing with respect to the amount of usage among younger ages [6,7]. Smoking and drinking in childhood and adolescence can be very deleterious, increasing the likelihood of risky behaviors (e.g., violent crimes and illicit drug use), damaging health, and leading to tobacco and alcohol dependence in adulthood [8,9]. Therefore, the exploration of the influencing factors of tobacco and alcohol use among children and adolescents has become a topic of wide concern. Although relevant studies have shown that cyber-victimization was correlated with adolescent tobacco and alcohol use, the underlying mechanism of this correlation remains largely unknown.

In addition, as one of the main socialization agents, peers (and especially deviant peers) represent a significant factor capable of influencing the problem behaviors of children and adolescents [10,11]. Furthermore, according to the enhancement model, affiliation with deviant peers may act as a moderator exacerbating the link between environmental risk factors and delinquency in childhood and adolescence [12]. Therefore, this study will further examine the moderating role of deviant peer affiliation on the association between cyber-victimization and tobacco and alcohol use. Meanwhile, potential age differences will also be tested among elementary, middle, and high school students. This may not only help us clarify the underlying moderating mechanism (i.e., “when” cyber-victimization impacts tobacco and alcohol use), but also provide intervention guidance for children and adolescents’ tobacco and alcohol use from the perspective of both peer interaction and age differences.

### 1.1. Cyber-Victimization and Tobacco and Alcohol Use

Smoking and drinking among children and adolescents have always been serious social concerns, and their influencing factors have been widely explored. It was found that stressful life events in one’s social environment associated with one’s family, school, and peers are important incentives for substance use [13]. In the current information age, stressful events in the cyberspace, such as being bullied, may also lead to problematic behaviors including tobacco and alcohol use among teenagers. Cyberbullying is a new form of bullying which has emerged from the rapid popularity of information communication technology [14]. Compared with traditional bullying, cyberbullying is characterized by anonymity, the ability to affect a large potential audience, and by being difficult to escape from [15,16], all of which may lead to more devastating outcomes for victims [4]. Cyber-victimization has become one of the most common interpersonal stressors for individuals, especially among adolescents [17].

According to general strain theory [18], negative stimuli (such as cyber-victimization) could cause individuals to fall into negative internal states (such as anger, anxiety) and further lead to negative coping styles. In this case, adolescents may turn to tobacco and alcohol use to cope with the negative feeling or stress caused by cyber-victimization [19]. In addition, the stress exposure model also suggests that stressful events will increase the risk for various health-related outcomes [17]. Emerging evidence has also revealed a positive relationship between cyber-victimization and individuals’ smoking, binge drinking, and general substance use [20,21,22]. Thus, it was hypothesized that cyber-victimization would be positively associated with tobacco and alcohol use (Hypothesis 1).

### 1.2. The Moderating Role of Deviant Peer Affiliation

At the same time, peers also play an important role in the development and adaptation of children and adolescents [23,24]. In particular, deviant peer affiliation is a risk factor contributing greatly to various problem behaviors such as fighting, smoking, and drinking [25,26]. Besides its direct effect on problem behavior, as an important social factor, deviant peer affiliation was also found to moderate the influence of other external risk factors (such as inter-parental conflict) on adolescent problem behavior [11].

Regarding the association between cyber-victimization and tobacco and alcohol use, affiliating with deviant peers may make victims develop a positive attitude regarding problem behaviors [27]. After being bullied online, victims with deviant peers are more likely to cope with this interpersonal pressure with negative behaviors such as smoking and drinking. In addition, according to the “risk-enhancing model” [28], one risk factor will enhance the negative effect of another risk factor on individuals’ adaptation. Therefore, we speculate that deviant peer affiliation would enhance the effect of cyber-victimization on adolescents’ tobacco and alcohol use. It was hypothesized that deviant peer affiliation would moderate the association between cyber-victimization and tobacco and alcohol use, with the association becoming stronger among individuals with more deviant peer affiliation (Hypothesis 2).

### 1.3. Age Difference

In addition, cyber-victimization and its association with internalizing and externalizing problems may be different for children and adolescents in different development periods. Firstly, regarding cyber-victimization, the rate of cyber-victimization an individual experiencing varies by grades. It increases from 6.4% in the sixth grade to 11.6% in the tenth grade, and then decreases again to 7.8% in the twelfth grade [29]. At the same time, some researchers think that older adolescents, who are high technology users [30], also have higher rates of substance use [31], which may in turn influence the relationship between cyber-victimization and substance use for adolescents at different ages.

Deviant peer affiliation, then, also has different shapes of developmental trajectories [12,26,32]. To be more specific, in pre-adolescence, mid-adolescence, and late adolescence, an individual’s deviant peer affiliation may be different. For example, researchers examined deviant peer affiliation among adolescents aged 12-16 years old and found that some adolescents who started with low deviant peer affiliation showed a slight increase over time [26]. Others also found that the probability of some adolescents to affiliate with deviant peers was near 0 at the age of 11. However, this rose quickly to a peak at age 15, and then begin declining afterwards [32]. These differences may also impact adolescent developmental outcomes including substance use [26]. At the same time, some researchers believe that due to the increase of time spent with peers, adolescents are more susceptible to peer influence and that peer influence is also stronger during adolescence [23]. Therefore, this study aims to further examine these potential age differences.

In conclusion, this study aims to examine the association between cyber-victimization and tobacco and alcohol use, as well as the moderating role of deviant peer affiliation. The potential age differences among elementary, middle, and high school students were also investigated.

## 2. Materials and Methods

### 2.1. Participants

Participants were recruited from two elementary schools (grades four, five and six), two middle schools (grades seven and eight) and two high schools (grades ten and eleven) in central China with the approval of the school authority and the Academic Committee for Scientific Research at the first author’s university. Through convenience sampling, a total of 1607 students participated in this study voluntarily. Surveys with a lack of gender and age information (*n* = 76), regular answers (*n* = 38), and over 30% of survey items were not answered (*n* = 5) were removed. The final usable sample consisted of 1488 students (52.6% boys), including 702 elementary school students (*M_age_* = 11.10, *SD* = 0.91; 51.2% boys), 318 middle school students (*M_age_* = 13.57, *SD* = 0.79; 45.6% boys), and 468 high school students (*M_age_* = 16.57, *SD* = 0.72; 58.3% boys).

### 2.2. Measurement

#### 2.2.1. Cyber-Victimization

The E-Victimization Scale (E-VS) [33] was adopted to assess Chinese adolescent cyber-victimization. Participants were asked to indicate the frequency of occurrences of certain situations to them in the last seven days prior to the survey on a 7-point scale (0 = *zero times*, 6 = *6 times or more*) on each of the six items, with higher scores indicating a higher frequency of cyber-victimization. In this study, the Cronbach’s alpha of this scale was 0.92 in the total sample and 0.94, 0.88, and 0.92 in the elementary, middle, and high school student samples, respectively.

#### 2.2.2. Deviant Peer Affiliation

The Chinese version of the deviant peer affiliation scale [34] was adopted. Participants were asked to indicate how many of their friends showed each of the eight deviant behaviors (e.g., smoking, alcohol use, aggression) during a period of six months on a 5-point scale (1 = *none*, 5 = *almost all*), with higher scores indicating more deviant peer affiliation. In this study, the Cronbach’s alpha of this scale was 0.84 in the total sample and 0.84, 0.86, and 0.84 in the elementary, middle, and high school student samples, respectively.

#### 2.2.3. Tobacco and Alcohol Use

The Chinese version of the tobacco and alcohol use scale [35] was used to measure the frequency and quantity of smoking and drinking in the past 30 days. Participants scored four items on a 6-point scale ranging from 1 = *Never* to 6 = *20–30 days* (or 1 = *Never* to 6 = *10 or more cigarettes/glasses per day*), with higher scores representing heavier tobacco and alcohol use. In this study, the Cronbach’s alpha of this scale was 0.83 in the total sample and 0.90, 0.81, and 0.79 in the elementary, middle, and high school student samples, respectively.

### 2.3. Ethics Approval

This study was approved by the Ethical Committee for Scientific Research at the researchers’ affiliated institution. The ethical values required in research with human beings, the fundamental principles included in the Helsinki Declaration (e.g., informed consent, protection of personal data, and guarantees of confidentiality), and the regulations of the education management department were followed. At the same time, all the participants and their parents were informed of the principles of the study, and parental consent for the children’s participation in the study was also obtained.

### 2.4. Statistical Analysis

All of the statistical analyses were conducted with the SPSS 23.0. First, the descriptive statistics and correlational analyses were calculated. Then, the PROCESS macro (http://www.afhayes.com, accessed on 11 February 2021) for SPSS (Model 1), suggested by Hayes [36], was adopted to test the moderating model with 5000 bias-corrected samples (the moderating effect is significant when 95% CI does not include zero) among elementary, middle, and high school students, respectively.

## 3. Results

### 3.1. Descriptive Statistics and Correlation Analysis

A Spearman correlation analysis was conducted. As presented in Table 1, there were significant positive correlations among cyber-victimization, deviant peer affiliation, and tobacco and alcohol use among elementary, middle, and high school students. Hypothesis 1 was supported.

### 3.2. Testing of Moderating Effect of Deviant Peer Affiliation

**Figure 1:**
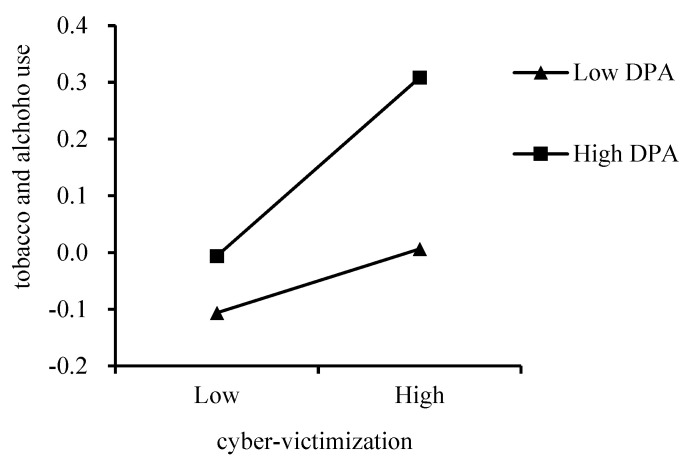
Moderating effect of deviant peer affiliation (DPA) on the relationship between cyber-victimization and tobacco and alcohol use among elementary school students.

The SPSS macro-Process (Model 1) was further conducted to test the moderating effect of deviant peer affiliation. As previous studies had indicated that gender was closely associated with both cyber-victimization and substance use [4], this was included in the analysis as a control variable. The results (Table 2) showed that, among elementary school students, cyber-victimization was significantly associated with tobacco and alcohol use (*β* = 0.11, *p* < 0.01). The interaction of cyber-victimization and deviant peer affiliation was also significantly associated with tobacco and alcohol use (*β* = 0.11, *p* < 0.001), indicating the significant moderating effect of deviant peer affiliation. Then, a simple slope test was conducted. As shown in Figure 1, although both slopes were significant, this association was stronger for elementary school students with higher deviant peer affiliation (*β_simple_* = 0.22, *p* < 0.001) than lower deviant peer affiliation (*β_simple_* = 0.08, *p* = 0.04). Thus, Hypothesis 2 was supported among elementary school students.

Regarding middle school students, neither cyber-victimization nor the interaction between cyber-victimization and deviant peer affiliation were significantly associated with tobacco and alcohol use. Thus, Hypothesis 2 was not supported among middle school students.

**Figure 2:**
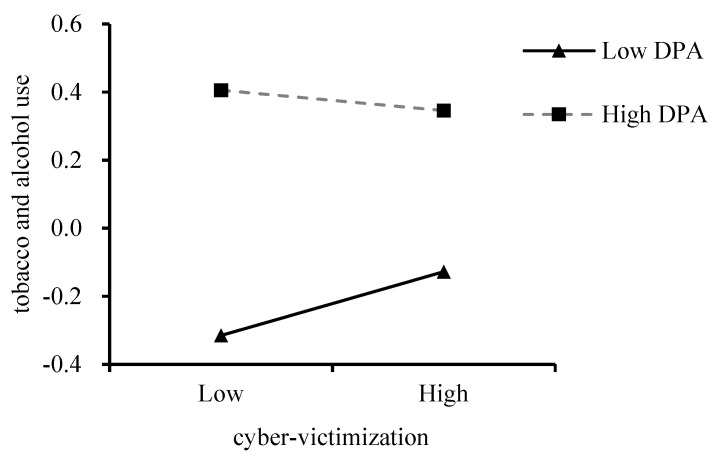
Moderating effect of deviant peer affiliation (DPA) on the relationship between cyber-victimization and tobacco and alcohol use among high school students. The solid black slope is significant (*p* < 0.05), the dashed grey slope is not.

Lastly, among high school students, the association between cyber-victimization and tobacco and alcohol use was non-significant, while the interaction between cyber-victimization and deviant peer affiliation was significantly associated with tobacco and alcohol use (*β* = −0.10, *p* < 0.01), indicating the significant moderating effect of deviant peer affiliation. Then, a simple slope test was conducted. As shown in Figure 2, the association between cyber-victimization and tobacco and alcohol use was significant for high school students with lower deviant peer affiliation (*β_simple_* = 0.13, *p* = 0.04), but was non-significant for high school students with higher deviant peer affiliation (*β_simple_* = −0.04, *p* = 0.34). This finding is contrary to the moderation’s direction of deviant peer affiliation in Hypothesis 2.

## 4. Discussion

Based on the general stress theory, previous empirical studies, and the realities of the daily life of children and adolescents, this study examined the association between cyber-victimization and tobacco and alcohol use, as well as the moderating role of deviant peer affiliation among elementary, middle, and high school students, respectively. The results found that cyber-victimization was significantly correlated with tobacco and alcohol use among elementary, middle, and high school students with different moderating mechanism. While deviant peer affiliation was found to act as a positive moderating effect in elementary school students and a negative moderating effect in high school students, this moderating effect was insignificant in middle school students.

Firstly, consistent with previous findings, our results showed that cyber-victimization was positively correlated with tobacco and alcohol use among elementary, middle, and high school students [22,27]. This may suggest that after being cyberbullied, children and adolescents may adopt negative or even self-medicating strategies (tobacco and alcohol use) to cope with the distress caused by victimization [37,38]. This is also in line with the general stress theory. Generally speaking, being cyberbullied brings victims great psychological and physical strain [39], and provides opportunity for negative coping strategies. At the same time, children and adolescents lack enough ability and appropriate strategies when dealing with stressful life events such as cyber-victimization due to their immaturity [40]. For example, they are usually reluctant to report their experience of victimization to teachers or parents for support [41]. As a result, being victimized on the internet would promote them to adopt maladaptive coping styles such as engaging in deleterious activities (e.g., drinking and smoking) to distract themselves from the stress and negative emotions.

Furthermore, we also found the association between cyber-victimization and tobacco and alcohol use was positively moderated by deviant peer affiliation among elementary school students. For pupils with more deviant peers, being cyberbullied was found to be associated with more tobacco and alcohol use. This may be because deviant peers provide the access to tobacco and alcohol. For elementary school students, apart from their family members, they may be less exposed to tobacco and alcohol, and deviant peers may represent one of their “windows” for exposure to cigarettes and alcohols [39]. The model role of deviant peers enhances their positive attitude towards and potential engagement in similar behaviors [27,42]. Therefore, when elementary school students who have affiliated with deviant peers experience cyber-victimization, they are likely to smoke and drink to cope with this interpersonal pressure. Moreover, affiliating with deviant peers may further increase the risk of being bullied or rejected for elementary school students [43], thereby reinforcing the incidence of problem behaviors.

Unexpectedly, for middle and high school students, although the correlation between cyber-victimization and tobacco and alcohol use was significant, the association between them was non-significant in the regression model. This is different from the results of some previous studies [4,19,20] and the results of the elementary school students in this study. First, tobacco and alcohol are thought to have different functions and may have different results; cyber-victimization was found to significantly relate to alcohol use but not to tobacco use in a mixed sample of middle and high school students [22]. Some other studies also found that cyber-victimization did not predict adolescent general substance use combined different types of substances (e.g., tobacco, alcohol, marijuana, cocaine, etc.) [44]. Second, this association may depend on other factors. For example, it was found that the association between cyber-victimization and substance use was stronger among adolescents with low levels of parental social support [4], poor relationship with their parents [19], and/or fewer family contacts [45].

In this study, although we did not find the moderating role of deviant peer affiliation in the association between cyber-victimization and tobacco and alcohol use among middle school students, it was found that this association was moderated by deviant peer affiliation among high school students. To our surprise, and contrary to our hypothesis, for high school students with high deviant peer affiliation, the association between cyber-victimization and tobacco and alcohol use was non-significant. For those with low deviant peer affiliation, the association between cyber-victimization and tobacco and alcohol use was positively significant. This result demonstrates that deviant peer affiliation did not amplify the adverse effect of cyber-victimization on high school students’ tobacco and alcohol use. Instead, it “weakened” the association between cyber-victimization and tobacco and alcohol use. This may be related to the gradual increase of peer effect during adolescence (that is, deviant peer affiliation plays a masking effect). For high school students in adolescence, with the increasing time spent with peers, peer relationships become the primary social context that influences social development and peer influence is stronger [46]. According to the socialization model, deviant peer affiliation is a sufficient cause of adolescent problem behaviors [12] (that is, deviant peer affiliation can directly lead to adolescent tobacco and alcohol use) [47]. Therefore, cyber-victimization contributes to tobacco and alcohol use among high school students with low deviant peer affiliation. However, when affiliating with more deviant peers, the direct link between deviant peer affiliation and tobacco and alcohol use was stronger. As a result, the adverse effect of cyber-victimization is masked and insignificant. In this study, we also found that high school students’ deviant peer affiliation was significantly higher than that of elementary school students (*M_high_* = 1.43; *M_ele_* = 1.17; *t* = 8.32, *p* < 0.001), and the correlation between deviant peer affiliation and tobacco and alcohol use was also significantly stronger among high school students (*r* = 0.41, *p* < 0.001). Although the moderating effect of deviant peer affiliation was not the “cumulative risk” effect we hypothesized among high school students, it is particularly troubling since the negative effect of cyber-victimization and deviant peer affiliation on high school students’ tobacco and alcohol use is more similar to a model in which “one trades and the other grows”.

### Limitationsand Implications

Several limitations should be acknowledged. Firstly, a cross-sectional design cannot draw causality. Particularly, some studies using longitudinal data have shown the causal relationship between cyber-victimization and tobacco and alcohol use [19,20], but others have failed to find the same result [44]. Therefore, various methods, including but not limited to longitudinal studies, can be used together in future to clarify this relationship. Secondly, this study only focused on the moderating role of deviant peer affiliation on the association between cyber-victimization and tobacco and alcohol use (as well as age differences). Future research needs to further investigate other possible internal mechanisms in this association. Thirdly, tobacco and alcohol use was not separate in this study, and future research may examine their influencing factors separately. Fourthly, the coincidence of substance use and experiencing cyber-victimization was not considered among the participants in this study.

The findings also have some implications. Theoretically, from the perspective of a peer context, this study further clarifies the boundary condition of the relationship between cyber-victimization and tobacco and alcohol use and also clarifies age differences. This deepens our understanding of the outcomes of cyber-victimization on children and adolescents and also contributes to revealing the underlying mechanism of cyber-victimization and tobacco and alcohol use. Practically, cyber-victimization, as a kind of negative experience in online interpersonal interaction, can induce children and adolescents’ offline problem behaviors. Therefore, children and adolescents themselves should use the internet healthily, learn to identify the security risks of information online, and timely ask parents or teachers to seek a reasonable way to deal with problems such as bullying. Moreover, parents should monitor children’s online behavior and teach them to know and deal with cyber-victimization; educators should carry out internet literacy and network security education, and cultivate students’ social skills and scientific interpersonal communication methods; and online media should develop information filtering and blocking functions to warn and block minors’ harmful information with intimidation and threats, so as to prevent bullying perpetration and bullying victimization. At the same time, parents and educators should also pay attention to the offline peer interaction of children and adolescents, especially to prevent them from interacting with delinquents. In addition, the differences among elementary, middle, and high school students should also be noticed so that educators can teach students in accordance with their aptitude in practical work.

## 5. Conclusions

To sum up, this study found that cyber-victimization was positively correlated with tobacco and alcohol use among elementary, middle, and high school students, and this relation was influenced by deviant peer affiliation. Among elementary school students, deviant peer affiliation positively moderated this relation. Among middle school students, deviant peer affiliation had no significant moderating role. And, lastly, among high school students, deviant peer affiliation negatively moderated this relation.

## Figures and Tables

**Table 1:** Descriptive statistics and correlations among study variables.

	Elementary			Middle			High		
Variables	1	2	3	1	2	3	1	2	3
1. CV	1			1			1		
2. DPA	0.16 ***	1		0.19 **	1		0.24 ***	1	
3. TAU	0.15 ***	0.18 ***	1	0.19 **	0.35 ***	1	0.13 **	0.41 ***	1
*M*	0.57	1.17	1.08	0.63	1.47	1.13	0.44	1.43	1.25
*SD*	1.32	0.52	0.40	1.17	0.67	0.45	1.07	0.54	0.59

Note, N_Ele_ = 702, N_Mid_ = 318, and N_High_ = 468. CV, cyber-victimization; DPA, deviant peer affiliation; TAU, tobacco and alcohol use. *** *p* < 0.001, ** *p* < 0.01.

**Table 2:** Testing the moderated effect among elementary, middle, and high school students.

Regression Equation			Fitting Index		Significance of Coefficients			
	Outcome	Predictors	*R^2^*	*F*	*β*	*SE*	LLCI	ULCI
Elementary	TAU		0.20	42.43 ***				
		Gender			−0.13	0.07	−0.26	0.01
		CV			0.11 **	0.04	0.04	0.18
		DPA			0.11 *	0.04	0.02	0.20
		CV × DPA			0.11 ***	0.02	0.07	0.14
Middle	TAU		0.03	2.82 *				
		Gender			−0.08	0.11	−0.30	0.14
		CV			0.03	0.06	−0.09	0.14
		DPA			0.18 **	0.06	0.06	0.29
		CV × DPA			−0.01	0.05	−0.10	0.08
High	TAU		0.20	28.21 ***				
		Gender			−0.34 ***	0.09	−0.52	−0.16
		CV			0.06	0.05	−0.04	0.15
		DPA			0.36 ***	0.05	0.27	0.45
		CV × DPA			−0.10 **	0.03	−0.15	−0.04

Note, Gender: boys = 1, girls = 2. CV, cyber-victimization; DPA, deviant peer affiliation; TAU, tobacco and alcohol use. *** *p* < 0.001, ** *p* < 0.01, * *p* < 0.05.
